# Momentary Successful Aging Indicators and Their Short-Term and Long-Term Covariates

**DOI:** 10.1093/geroni/igaa057.2081

**Published:** 2020-12-16

**Authors:** Dwight Tse, Kelsey Finley, Katherine Vrooman

**Affiliations:** 1 The Chinese University of Hong Kong, Hong Kong, China; 2 Claremont Graduate University, Claremont, California, United States

## Abstract

Studies on successful aging have conceptualized it as a between-person construct, meaning that people’s aging process is seen as more or less successful than others’ across contexts. This study examines within-person, moment-to-moment successful aging indicators, such as (absence of) physical pain, good physical and cognitive functioning, and active engagement in social and productive activities, and their relations to one-time well-being indicators (affective balance, psychological needs satisfaction, meaning in life, and satisfaction with life) at the time and one year after. Multilevel modeling on experience sampling data revealed that successful aging varied substantially (over 50% total variances) within-person and was positively associated with well-being. This study illustrates the utility of momentary successful aging indicators and advocates for a more nuanced understanding besides a simple classification of older adults undergoing “successful” or “normal” aging processes.

